# The Changing Epidemiology of Bloodstream Infections and Resistance in Hematopoietic Stem Cell Transplantation Recipients

**DOI:** 10.4274/tjh.2014.0378

**Published:** 2016-08-19

**Authors:** Mücahit Yemişen, İlker İnanç Balkan, Ayşe Salihoğlu, Ahmet Emre Eşkazan, Bilgül Mete, M. Cem Ar, Şeniz Öngören, Zafer Başlar, Reşat Özaras, Neşe Saltoğlu, Ali Mert, Burhan Ferhanoğlu, Recep Öztürk, Fehmi Tabak, Teoman Soysal

**Affiliations:** 1 İstanbul University Cerrahpaşa Faculty of Medicine, Department of Infectious Diseases and Clinical Microbiology, İstanbul, Turkey; 2 İstanbul University Cerrahpaşa Faculty of Medicine, Department of Internal Medicine, Division of Heamatology, İstanbul, Turkey; 3 Koç University Faculty of Medicine, American Hospital, Clinic of Internal Medicine, Division of Heamatology, İstanbul, Turkey

**Keywords:** Hematopoietic stem cell transplantation, Bloodstream infection, Epidemiology, resistance, Central venous catheter

## Abstract

**Objective:**

Patients receiving hematopoietic stem cell transplantation (HSCT) are exposed to highly immunosuppressive conditions and bloodstream infections (BSIs) are one of the most common major complications within this period. Our aim, in this study, was to evaluate the epidemiology of BSIs in these patients retrospectively.

**Materials and Methods:**

The epidemiological properties of 312 patients with HSCT were retrospectively evaluated.

**Results:**

A total of 312 patients, followed between 2000 and 2011, who underwent autologous (62%) and allogeneic (38%) HSCT were included in the study. The most common underlying malignancies were multiple myeloma (28%) and Hodgkin lymphoma (21.5%). A total of 142 (45%) patients developed at least 1 episode of BSI and 193 separate pathogens were isolated from the blood cultures. There was a trend of increase in the numbers of BSIs in 2005-2008 and a relative increase in the proportion of gram-positive infections in recent years (2009-2011), and central venous catheter-related BSI was found to be most common source. Coagulase-negative staphylococci (49.2%) and Acinetobacter baumannii (8.8%) were the most common pathogens. Extended-spectrum beta-lactamase-producing strains were 23% and 22% among Escherichia coli and Klebsiella spp. isolates, respectively. Quinolone resistance was detected in 10% of Enterobacteriaceae. Resistance to carbapenems was not detected in Enterobacteriaceae, while it was seen at 11.1% and 23.5% in Pseudomonas and Acinetobacter strains, respectively.

**Conclusion:**

A shift was detected from gram-negative bacteria to gram-positive in the etiology over the years and central lines were the most common sources of BSIs.

## Abstract (tr)

**Amaç:**

Hematopoetik kök hücre transplantasyonu (HKHT) yapılan hastaların bağışıklık sistemi ciddi şekilde baskılanmıştır ve kan akımı enfeksiyonları (KAE) bu süre içinde karşılaşılan majör komplikasyonlardan biridir. Bu çalışmada amacımız, geriye dönük olarak bu hastalarda KAE’lerinin epidemiyolojisini değerlendirmektir.

**Gereç ve Yöntemler:**

HKHT yapılan 312 hastanın epidemiyolojik özellikleri retrospektif olarak değerlendirildi.

**Bulgular:**

2000 ve 2011 yılları arasında otolog (%62) ve allojeneik (%38) HKHT yapılan 312 hasta, çalışmaya dahil edildi. Çalışmaya dahil edilen hasta grupları en sık multipl miyelom (%28) ve Hodgkin lenfoma (%21,5) tanılı hastalar idi. Yüz kırk iki hastada (%45) en az bir kez KAE gelişmiş ve kan kültürlerinden 193 ayrı patojen elde edilmiştir. KAE’lerde 2005-2008 yılları arası bir artışın yanında, 2009-20011 yılları arasında da gram pozitiflerde göreceli bir artış da saptanmış ve en sık KAE kaynağı santral venöz kataterler olarak tespit edilmiştir. Koagülaz negatif staphylococi (%49,2) ve Acinetobacter baumannii (%8,8), kan kültürlerinden en sık elde edilen patojenlerdir. Genişlemiş spektrumlu beta laktamaz üretimi Escherichia coli ve Klebsiella spp. suşları arasında sırası ile %23 ve %22 idi. Kinolon dirençli Enterobacteriaceae oranı %10 olarak tespit edilmiştir. Pseudomonas ve Acinetobacter suşlarında karbapenem direni sırasıyla, %11,1 ve %23,5 iken, Enterobacteriaceae grubunda karbapenemlere hiç direnç saptanmamıştır.

**Sonuç:**

Yıllar içinde, gram negatif bakterilerden gram pozitiflere doğru bir kayma gözlenirken, en sık KAE kaynağı santral kataterler olarak saptanmıştır.

## INTRODUCTION

Bloodstream infection (BSI) is the most common infectious problem in patients undergoing hematopoietic stem cell transplantations (HSCTs). Depending on the protocol used for transplantation and the duration of neutropenia, approximately 13%-60% of patients develop BSIs, which can result in delays in chemotherapies, extension of admission period, and increased costs of antimicrobial therapy against target organisms [1,2]. The differences in results of these studies are probably due to different study designs, study populations, conditioning regimens, and prophylactic antibiotic protocols [1]. Beside neutropenia, the other risk factors for BSI include age, underlying disease, presence of a central catheter, severe graft-versus-host disease (GVHD), mucositis, and steroid use [1,3,4].

The etiology of BSIs has changed and showed different patterns in the past years. While gram-negative BSIs among neutropenic cancer patients were formerly the leading cause of bacteremia, the etiology of BSIs in this patient population has become predominantly gram-positive, and especially viridans group streptococci and coagulase-negative staphylococci, over the last 2 decades [5,6]. Besides this shift, resistance rates and patterns also started to change and more resistant microorganisms are now found as the causes of BSIs. For example, the emergence of fluoroquinolone-resistant bacteria, increase in multidrug-resistant gram-negative bacteria, increase in nosocomial methicillin-resistant Staphylococcus aureus infections, and emergence of extended-spectrum beta-lactamase (ESBL) producers have all been reported in the literature in neutropenic patients [3]. Due to the diversity of the causative microorganisms of BSIs in patients with HSCT, information about etiology and antibiotic susceptibility of BSIs is important to initiate effective antibiotic treatment, a parameter that has been shown to be closely associated with survival in bacteremic patients [7]. In this study, we aimed to assess the etiology and clinical characteristics of BSIs in patients with hematological malignancies undergoing HSCT over a 12-year period. We also evaluate the risk factors, resistance patterns, and sources of BSIs in this group of patients as a secondary objective.

## MATERIALS AND METHODS

### Patients

A total of 312 patients who underwent autologous and allogeneic bone marrow transplantation in the Stem Cell Transplantation Unit of the İstanbul University Cerrahpaşa Medical School from 1 January 2000 to 31 December 2011 were included in the study. Data on demographic features of the patients, underlying disease, disease status prior to HSCT, HSCT protocols, prophylaxis regimens, and emerging resistance profiles of bacteremia were retrospectively analyzed. The data of the patients were recorded from the initiating day of conditioning until the 100th day after transplantation.

### Hematologic Definitions

All patients were followed in isolated single rooms equipped with high-efficiency particulate air filters and underwent central venous catheter (CVC) insertion. Conditioning was done using standard protocols such as cyclophosphamide alone or in combination with total body irradiation for allogeneic transplantation and CBV (cyclophosphamide, VP-16, BCNU) or BEAM (BCNU, VP-16, cytarabine, melphalan) for autologous stem cell transplantation. Almost all allogeneic transplantations were done from HLA-identical sibling or matched unrelated donors. Neutrophil engraftment was defined as the first of 3 consecutive days on which the absolute neutrophil count remained at or above 500/mm^3^ after stem cell infusion. GVHD diagnosis and staging were performed according to previously established criteria [8,9].

### Microbiological Definitions

We obtained at least 2 blood cultures from all febrile neutropenic patients and initiated an antipseudomonal antibiotic. Febrile neutropenia was investigated and managed according to the Infectious Disease Society of America guidelines [10,11].

BSI (mono or poly) and catheter-associated BSI were accepted according to the established criteria [12,13,14]. Antimicrobial susceptibility tests of bacteria obtained from blood cultures were evaluated by the disk diffusion method according to the current Clinical and Laboratory Standards Institute (CLSI, formerly NCCLS) criteria [15]. Intermediate sensitivity or resistance results were accepted as resistant. The screening of multidrug-resistant phenotypes including methicillin-resistant Staphylococcus aureus, ampicillin- and vancomycin-resistant enterococci, ESBL production, and carbapenemase production was conducted according to CLSI recommendations [16,17]. Multidrug resistance was defined as acquired nonsusceptibility to at least 1 agent in 3 or more antimicrobial categories; extensive drug resistance was defined as nonsusceptibility to at least 1 agent in all but 2 or fewer antimicrobial categories, and pandrug resistance was defined as nonsusceptibility to all agents in all antimicrobial categories [18].

### Statistical Analysis

The categorical data were compared by chi-square tests, and p<0.05 was accepted as significant. Factors predicting bacteremia and mortality were analyzed by logistic regression analyses.

## RESULTS

A total of 312 patients were included in the study. The number of female patients was 137 (44%) and the mean age was 39 years (minimum-maximum: 12-73 years). The most common underlying conditions of the patients were multiple myeloma in 87 (28%) and Hodgkin lymphoma in 67 (21.5%). The number of patients who underwent autologous and allogeneic HSCT was 194 (62%) and 118 (38%), respectively. The stem cell source was peripheral blood in 295 (94.5%) patients and bone marrow in 17 (5.5%) patients. The mean time to neutrophil engraftment was 14 days and the number of patients having detectable cytomegalovirus-DNA was 38 (12.2%). The number of patients having acute GVHD equal to or above stage 2 was 36 (11.5%). Table 1 shows the characteristics of the patients.

We obtained a total of 193 microbial isolates from patients’ blood cultures; of these 193 isolates, 12 were obtained after the conditioning regimen (before infusion of cells), 140 were obtained after infusion of cells (before neutrophil engraftment), and 41 were obtained after engraftment. Table 2 shows the properties of the isolates obtained from blood cultures. Gram-positive, gram-negative, and fungal isolates obtained from the blood cultures were 112 (58%), 74 (38.3%), and 7 (3.7%), respectively. A total of 142 (45.5%) of 312 patients developed at least 1 episode of BSI. Of these 142 patients, 68 had autologous and 74 had allogeneic HSCT. In our study, 106 patients developed 1 episode of BSI, 32 patients had 2 episodes, 3 patients had 3 episodes, and 1 patient had 4 episodes. The numbers of monomicrobial and polymicrobial episodes were 168 and 14, respectively. The source of BSI was determined as CVC-associated for 151 (78.2%) isolates while no source could be determined for the remaining isolates. Of those 151 CVC-associated isolates, 69.5% were gram-positive bacteria.

The most frequently isolated gram-positive bacteria were coagulase-negative staphylococci with 95 isolates, and then Streptococcus spp. with 8, S. aureus with 5, Enterococcus spp. with 2, and gram-positive rods with 2 isolates. The numbers of gram-negative isolates obtained from blood cultures were as follows: Acinetobacter baumannii, 17; Stenotrophomonas maltophilia, 14; Escherichia coli, 13; Klebsiella spp., 9; Pseudomonas aeruginosa, 9; and other gram-negative bacteria, 12 isolates. A total of 7 fungal isolates comprised 3 Candida parapsilosis, 1 Candida tropicalis, 1 Fusarium spp., and 2 Candida spp. isolates.

**Figure 1:**
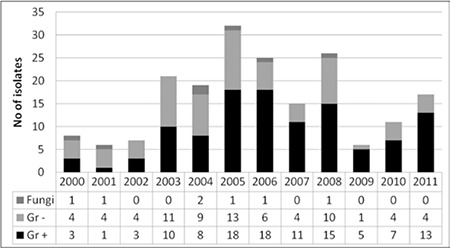
Evolution of bloodstream infection etiology.

Between 2000 and 2005, the number of gram-negative isolates was greater than the number of gram-positive isolates; after 2005, gram-positive isolates increased in frequency and became the major causative group for BSIs. Figure 1 shows the etiology of BSIs (gram-positive, gram-negative, and fungal) according to year; there was a trend of increase in the numbers of BSIs in 2005-2008 and also a relative increase in the proportion of gram-positive BSIs in more recent years (2009-2011).

The number of ESBL-producing Enterobacteriaceae isolates was 6 (20.6%). Among the 13 E. coli isolates, 3 were ESBL-producing and 4 were resistant to ciprofloxacin, 5 to aminoglycosides, 2 to cefepime, and 3 to third-generation cephalosporin and piperacillin/tazobactam. For Klebsiella spp., 2 isolates were resistant to third-generation cephalosporins, 3 to piperacillin/tazobactam, and 2 to cefepime, and 2 were ESBL-producing. We found no resistant isolates for aminoglycosides or ciprofloxacin. The proportion of isolates that were ESBL-producing among E. coli and Klebsiella spp. was 23% and 22%, respectively. No resistance to carbapenems was observed.

In the P. aeruginosa group, 2 strains were resistant to ceftazidime; 1 was resistant to piperacillin/tazobactam, cefepime, and carbapenems; and no strains were resistant to aminoglycosides or ciprofloxacin. Of the 17 A. baumannii strains, 4 were resistant to carbapenems; 3 to aminoglycosides, ceftazidime, and cefepime; 2 to piperacillin/tazobactam; and 1 to ciprofloxacin. All S. maltophilia strains were susceptible to trimethoprim/sulfamethoxazole. Among all gram-negative strains, the rate of multidrug-resistant bacteria was 12.1%, and the rate of extensively drug resistant bacteria was 8.1%. We did not identify any pandrug resistance in our study. Table 3 shows the resistance patterns of the gram-negative bacteria obtained from blood cultures.

Among all gram-positive bacteria, 95 (84.8%) were coagulase-negative staphylococci, and only 3 (3.1%) strains were susceptible to methicillin. In 5 S. aureus strains, only 1 was resistant to methicillin, and the remaining were susceptible. Among the 8 Streptococcus strains isolated, 7 were viridans group streptococci and 1 was group A beta-hemolytic streptococcus.

Univariate analysis to determine risk factors for bacteremia identified HSCT type, any comorbidity, duration of engraftment longer than 10 days, and GVHD grade of 2-4 (p<0.05). In multivariate analyses, only the type of HSCT (allogeneic) was associated with bacteremia (p<0.05).

The crude death rate in the 100 days after transplantation was 12.8%. Univariate analysis of risk factors for mortality revealed association with type of HSCT, presence of bacteremia, degree of GVHD, and engraftment period longer than 10 days (p<0.05). Only allogeneic HSCT was associated with mortality in multivariate analysis (p<0.05). The highest mortality rate was observed in the patients who had bacteremia due to S. maltophilia.

## DISCUSSION

Bloodsteam infections remains the main challenge for patients undergoing HSCT. Cappellano et al. reported the rate of bacteremia as 27% in 315 allogeneic HSCT patients [19]. In the studies of Poutsiaka et al. and Mikulska et al., the rates of bacteremia were found to be 43.6% and 38.4%, respectively [1,20]. In our study the rate of bacteremia was 45.5% among 312 HSCT patients.

Several features of the microorganisms obtained from the blood cultures of our patients changed over the study period. Between 2000 and 2005, the isolates of BSIs were predominantly gram-negative. After 2005, parallel to similar reports, this pattern switched to a gram-positive predominance, the majority of isolates being coagulase-negative staphylococci. As indicated in previous reports, this can be explained by the different conditioning regimens used for transplantation, antibiotic prophylaxis, or changing of global bacterial resistance [3]. Coagulase-negative staphylococci are usually the most frequently isolated gram-positive bacteria, while other gram-positive bacteria such as Enterococcus spp. have been reported to be more frequent in other studies [19,21,22]. In our study, the number of gram-positive bacteria other than coagulase-negative staphylococci was limited, so those resistance rates were not taken into account.

In the present study, A. baumannii and S. maltophilia isolates were predominant among gram-negative bacteria. Most bacteremia cases in our unit due to A. baumannii and S. maltophilia were found to be associated with insertion of central catheters. However, after 2007, only one case of bacteremia associated with these pathogens was reported. This change may be explained by the establishment of a team at our center for placement of central catheters in 2007. After the implementation of that team, the rates of bacteremia due to these two isolates sharply decreased. However, the rate of BSIs due to coagulase-negative staphylococcus is still high. The association between S. maltophilia and central catheters in HSCT patients was reported in some studies: in the study by Chaplow et al., an outbreak of S. maltophilia was found to be associated with CVCs [23]. In another study, Williamson et al. reported the source of bacteremia due to nonfermentative gram-negative bacteria to be a consequence of central catheters [24]. However, Labarca et al. attributed mucositis rather than central catheterization to be the source of S. maltophilia [25].

ESBL production among Enterobacteriaceae was 20.6% in the study group, while it was around 30% in other patients at our hospital; Mikulska et al. found it to be over 40% among HSCT patients [20]. Carbapenemase-producing Enterobacteriaceae have not been observed in any of our patients. However, recently we had a carbapenemase-producing Klebsiella (CPK) outbreak in neutropenic patients in our hematology unit, and Zuckerman et al. also reported an outbreak of CPK in HSCT patients [26].

Only 10% of the gram-negative bacteria were resistant to ciprofloxacin in the study. This rate is lower than that noted in other units of our hospital and those in other studies [3,20,27,28]. Busca et al. reported that quinolone use may induce the resistance rate against quinolone itself and even against some other antibiotics [28]. However, despite routine use of quinolone prophylaxis, increased rates of resistance have not been observed in patients undergoing prophylaxis.

In previous reports, BSI in patients who had undergone HSCT was generally found to be associated with age, late stage of underlying disease, GVHD, steroid use, mucositis, and central catheters [1,3,29]. In our study, we only identified central catheters as a source of BSI. In 93.75% of gram-positive bacteremia and 54.7% of gram-negative cases, the source was found to be associated with the presence of a CVC. In a few studies, the main source of BSI in HSCT patients was CVC-associated; Yuen et al. reported the rate of CVC-associated BSI to be 38% in the postengraftment period [30]. The rate of CVC-associated BSI was found to be 23% in the study of Liu et al [3]. In our series, the leading etiology was coagulase-negative staphylococci (95/193; 49%). In all of these patients the suggested source was a CVC. In gram-negative cases, however, CVCs were considered as the source in nearly half of the cases.

In multivariate analysis, bacteremia and death were found to be associated with the type of transplantation (allogeneic). We were not surprised by this result, because allogeneic transplantation is a highly immunosuppressive condition and other factors such as GVHD, immunosuppressive treatment for GVHD, long duration of neutropenia, and long time for engraftment are also associated with allogeneic transplantation. In our study, the mortality rate of patients having bacteremia due to S. maltophilia was found to be higher than with other bacteria. This may be due to the lack of activity of initial antibiotic treatment against S. maltophilia.

Our study had some limitations. It was performed retrospectively and, due to missing data, some patients had to be excluded. The initial empiric antibiotic treatment might have influenced the resistance of the bacteria, but we could not account for the effect of empiric antibiotic treatment. The impact of the stem cell source on bacteremia in allogeneic HSCT recipients could not be analyzed since almost all patients received peripheral stem cells from fully matched donors.

In conclusion, BSI in HSCT recipients is still a great problem. The global switch from a gram-negative etiology to a gram-positive one was also observed in our study. In addition to other gram-negative bacteria, A. baumannii and S. maltophilia were frequent causes of bacteremia but were generally not covered by initial empirical therapy. Accordingly, we observed a higher rate of mortality due to S. maltophilia bacteremia. It is generally difficult to identify the source of bacteremia in HSCT patients. In our study, CVCs were the only source suggested and they were usually associated with unusual pathogens. However, with a dedicated CVC team and the use of a catheter-care bundle, we could reduce the rate of catheter-related BSIs. HSCT recipients are especially at risk of CVC-related BSIs, which may include difficult-to-treat pathogens.

## Ethics

Ethics Committee Approval: Retrospective study; Informed Consent: It was taken.

## Figures and Tables

**Table 1:**
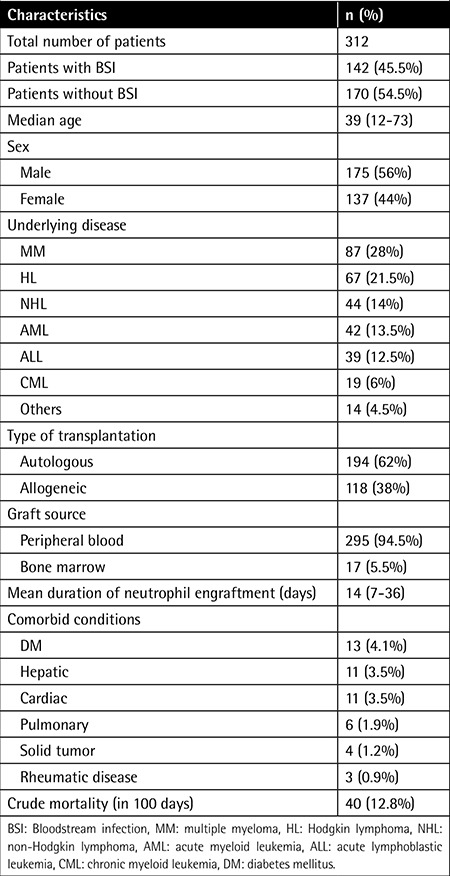
Characteristics of the patients.

**Table 2:**
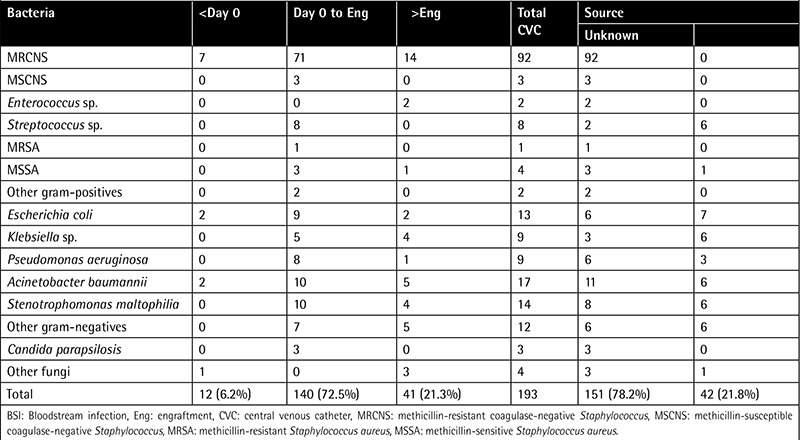
Etiology and source of bloodstream infections.

**Table 3:**
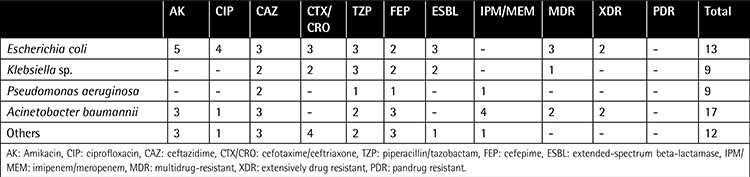
Resistance pattern of gram-negative isolates.
